# Impact of renal dysfunction on the choice of diagnostic imaging, treatment strategy, and outcomes in patients with stable angina

**DOI:** 10.1038/s41598-019-44371-4

**Published:** 2019-05-27

**Authors:** Takao Kato, Yukari Uemura, Masanao Naya, Mitsuru Momose, Naoya Matsumoto, Eriko Suzuki, Satoshi Hida, Takatomo Nakajima, Takao Yamauchi, Nagara Tamaki

**Affiliations:** 1 Department of Cardiovascular Medicine, Graduate School of Medicine, Kyoto University, Kyoto, Japan; 2 Biostatistics Division, Clinical Research Support Center, University of Tokyo Hospital, Tokyo, Japan; 3 Department of Cardiovascular Medicine, Hokkaido University Graduate School of Medicine, Sapporo, Japan; 4 Department of Diagnostic Imaging and Nuclear Medicine, Tokyo Women’s Medical University, Tokyo, Japan; 5 Department of Cardiology, Nihon University Hospital, Tokyo, Japan; 6 Department of Nuclear Medicine, Hokkaido University Graduate School of Medicine, Sapporo, Japan; 7 Department of Cardiology, Tokyo Medical University, Tokyo, Japan; 8 Saitama Cardiovascular and Respiratory Center, Kumagaya, Japan; 9 Cardiovascular medicine, Japan Community Health care Organization Sagamino Hospital, Sagamihara, Japan

**Keywords:** Interventional cardiology, Outcomes research

## Abstract

We investigated the interaction between the prognostic impact of a decrease in eGFR and the choice of initial diagnostic imaging modality for coronary artery disease. Out of 2878 patients who enrolled in the J-COMPASS study, 2780 patients underwent single photon emission computed tomography (SPECT), coronary computed tomography (CT) angiography, or coronary angiography (CAG) as an initial diagnostic test. After excluding patients with routine hemodialysis or lacked serum creatinine levels, 2096 patients in the non-decreased eGFR group (eGFR ≥ 60 ml/min/1.73 m^2^) and 557 patients in the decreased eGFR group (eGFR < 60 ml/min/1.73 m^2^) were analyzed in this study. Major adverse cardiac events, including death, myocardial infarction, heart failure hospitalization, and late revascularization, were followed, with a median follow-up duration of 472 days. SPECT or CAG was preferable to CT in patients in the decreased eGFR group (p < 0.0001 and p = 0.0024, respectively). There was a marginally significant interaction between the prognostic impact of a decrease in eGFR and the choice of diagnostic imaging modality (interaction-p = 0.056). A decrease in eGFR was not associated with a poor outcome in patients who underwent CT, while a decrease in eGFR was associated with poor outcomes in patients who underwent SPECT or CAG. In conclusion, the prognostic impact of a decrease in eGFR tended to be different among the initial imaging modalities.

## Introduction

Chronic kidney disease (CKD) caused more deaths worldwide in 2015 than in 2005^1^. Patients with CKD most commonly die from cardiovascular diseases^2,3^. Furthermore, the presence of CKD has a negative impact on the short- and long-term prognoses of cardiovascular diseases^2^. Positive findings of ischemia are more prevalent with a decline in creatinine clearance^4^.

It is important to choose the appropriate diagnostic imaging modality to detect coronary artery disease (CAD) in symptomatic patients with suspected CAD. Although the superiority of anatomical testing when compared with functional testing has long been debated, Douglas *et al*. reported that a strategy of anatomical testing with CTA, did not reduce the incidence of MACE as compared with functional testing^5^. However, there were no data available on renal function in the PROMISE trial^5^. In patients with renal dysfunction, the use of the contrast-enhanced medium should be minimal. In addition, comorbidities accelerate atherosclerosis, leading to calcification of the coronary artery. Thus, the choice of imaging modality in patients with renal dysfunction may be different from that in patients with normal renal function. Coronary computed tomography (CT) angiography, myocardial perfusion imaging (MPI), and coronary angiography (CAG) are three major imaging modalities used to diagnose CAD in patients with angina. There are many studies revealing the utility of each of these diagnostic imaging modalities in patients with renal dysfunction^6–14^. However, there have been no comparisons among these modalities, nor any data on the choice of modality in patients with renal dysfunction. The Japanese Coronary-Angiography or Myocardial Imaging for Angina Pectoris Study (J-COMPASS) Multicenter Study^15^, a study with a non-random and physician-referred design, reported that the choice of initial imaging modality was linked to the subsequent revascularization therapy and risk of major adverse cardiac events (MACE) at one year in symptomatic patients with CAD. In the original study, the use of single photon emission computed tomography (SPECT) and CT was associated with a lower risk of MACE than was the use of invasive CAG; however, the use of CT and CAG was associated with more frequent elective revascularization^15^.

In this sub-study, we sought to test whether all three initial diagnostic tests for CAD (CT, SPECT, and CAG) are associated with MACE for patients with a decrease in eGFR. We also aimed to see if there is any difference in the treatment strategy by a different type of initial diagnostic test between patients with and without a decrease in eGFR.

## Methods

### Patients

The design and main trial results of the J-COMPASS study have been published previously^15^. A total of 2,878 consecutive patients with suspected stable angina, from 81 centers in Japan with high-end diagnostic facilities, were enrolled. On the basis of the results of the initial tests and other clinical findings, well-trained cardiologists determined the initial diagnostic imaging modality to be used and the treatment strategy. Among these patients, 2780 patients who underwent SPECT, CT, or CAG as an initial diagnostic test and who had been routinely followed up were analyzed in the J-COMPASS study^15^. Symptomatic patients advised to undergo SPECT, CT, or CAG as the initial diagnostic test for suspected chronic CAD were enrolled. All patients underwent either stress SPECT (n = 1205), CT (n = 625), or CAG (n = 950) as an initial test for the diagnosis of CAD. The exclusion criteria of the original study were acute coronary syndrome at presentation or within a short period after the initial test, and a history of myocardial infarction (MI) or revascularization therapy.

**Figure 1:**
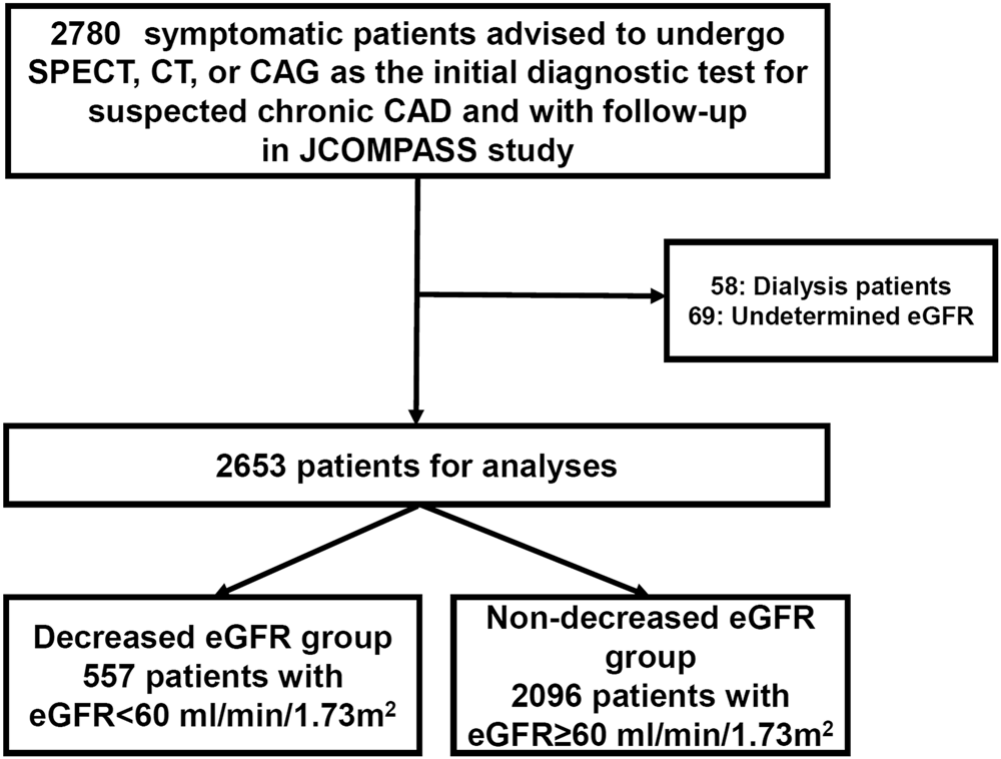
Patient flowchart. SPECT = single photon emission computed tomography, CT = computed tomographic angiography, CAG = coronary angiography, CAD = coronary artery disease, J-COMPASS = Japanese Coronary-Angiography or Myocardial Imaging for Angina Pectoris Study, eGFR = estimated glomerular filtration rate.

In this sub-study, we excluded patients who had undergone routine hemodialysis (n = 58) or lacked data on the serum creatinine levels (n = 69). Thus, the final study population included 2653 patients (Fig. 1 and Supplementary Table 1). We calculated the estimated glomerular filtration rate (eGFR) as follows: eGFR (ml/min/1.73 m^2^) = 194 × Cr^−1.094^ × (Age)^−0.287^ in men and 194 × Cr^−1.094^ × (Age)^−0.287^ × 0.739 in women^16^, and classified patients according to their eGFR. We analyzed patients with stage 2 CKD or lower (eGFR ≥ 60 ml/min/1.73 m^2^; non-decreased eGFR group) and stage 3 CKD or higher (eGFR < 60 ml/min/1.73 m^2^; decreased eGFR group). Comorbidities were based on the physician’s evaluation. Cerebrovascular disease was defined by stroke or vascular disease requiring the intervention by a neurosurgeon. Malignancy was defined according to various cancers and hematologic neoplasm.

### Treatment strategy and outcome measures

On the basis of the results of the initial tests and other clinical findings, the physicians chose the treatment strategy^15^. The treatment strategies included (1) medical therapy, which indicated medical therapy with same medication at the same dose after the initial test; (2) escalation of medical therapy, which indicated an increase in the dose of the same medication or introduction of new medication, and (3) intervention and escalation of medical therapy. The end-point was MACE: death, acute MI, heart failure hospitalization and late revascularization (>3 months) in accordance with the original study^15^.

### Definition of obstructive CAD in coronary CTA and CAG and functionally significant result in SPECT

We adopted the definitions used by the J-COMPASS study^15^. On CTA or CAG, patients with 1 or more diseased vessel (>50% diameter stenosis in segment 5, 6, 7, 11, 13, 1, 2, or 3) were considered to have a significant stenosis^17,18^. For the SPECT group, SPECT images were divided into 17 segments, each of which was scored five points under both stress and rest conditions (0, normal; 1, mildly reduced; 2, moderately reduced; 3, severely reduced; 4, absent) according to the American Heart Association criteria^19^ and summed stress score (SSS) ≥ 2 was considered a functionally significant result^20^.

### Ethics

All methods were carried out according to the principles of the Declaration of Helsinki. The study protocol was approved by the institutional review board of each participating center (Appendix). All participating patients provided written/oral informed consent before study enrollment^15^. We anonymized the patient record/information before analysis.

### Statistical analysis

In the present analysis, (1) we compared the baseline characteristics of patients belonging to the non-decreased eGFR and decreased eGFR groups, (2) we investigated whether a decrease in eGFR affects the selection of the initial diagnostic modality and treatment, and (3) we compared the outcome measures between the two patient groups and tested the interaction of its impact on prognosis and modalities.

Categorical variables were expressed as numbers and percentages and were compared using a chi-square test. Continuous variables were expressed as means (standard deviation [SD]) or median and interquartile range [IQR]. Continuous variables were compared using Student’s t-test between 2 groups and one-way analysis of variance among 3 groups.

To analyze the factors associated with the initial diagnostic modalities, we used a multinomial logistic regression model involving the following 13 potential independent, clinically relevant variables: age ≥60 years; sex; body mass index; presence of hypertension, dyslipidemia, diabetes, hyperuricemia, chronic obstructive pulmonary disease, and aortic disease (aneurysm or dissection); Canadian Circulation Society class 2 or higher^21^; current smoking; New York Heart Association (NYHA) functional class 2 or higher^22^; and a decrease in eGFR (Table 1). The adjusted odds ratios (ORs) relative to the choice of CT and 95% confidence intervals (CIs) were calculated. We set the choice of CT as a reference because the proportion of decrease in the eGFR was small. Next, to analyze the factors associated with the treatment strategies, we also used a multinomial logistic regression model including 15 variables: that is, the above-mentioned 13 variables and diagnostic modalities. When assessing the diagnostic modalities, we set SPECT as a reference according to the original J-COMPASS study^15^. Third, the Kaplan–Meier method was used to estimate the MACE rate between the decreased or non-decreased eGFR groups; the log-rank test was used for univariate comparisons. To compare the risks between the decreased or non-decreased eGFR groups, a multivariable Cox proportional hazard model was developed for MACE. The results are expressed as hazard ratios (HRs) and 95% confidence intervals (CIs). We selected 13 clinically relevant risk-adjusting variables, as mentioned above. Subgroup analyses for MACE were also performed with each diagnostic modality. Finally, we tested the interaction between the prognostic impact of renal dysfunction and each diagnostic modality. As a Supplementary Analysis, we have analyzed the MACE rates in the CT, SPECT, and CAG groups among patients with renal dysfunction using the Kaplan–Meier method.

**Table 1:** Patient characteristics.

		Non-decreased eGFR group		Decreased eGFR group		p value
		(n = 2096)		(n = 557)		
Age (years)		65.47	10.43	70.87	8.94	**<0.0001**
Age ≥60 years old*		1476	70.4%	493	88.5%	**<0.0001**
Female*		888	42.4%	211	37.9%	0.056
Height (cm)		159.32	8.99	158.8	8.99	0.23
Weight (Kg)		60.98	11.71	61.51	10.64	0.33
BMI (kg/m^2^)*^,||^		23.92	3.49	24.33	3.32	**0.014**
Systolic BP (mmHg)		137.09	19.29	137.96	19.49	0.35
Diastolic BP (mmHg)		78.2	11.97	75.8	11.72	**<0.0001**
Smoking*		520	24.8%	128	23.0%	0.37
Hypertension*		1149	54.8%	386	69.3%	**<0.0001**
Dyslipidemia*		1006	48.0%	259	46.5%	0.53
Diabetes*		584	27.9%	181	32.5%	**0.032**
Hyperuricemia*		99	4.7%	50	9.0%	**0.0001**
Familial history of CAD		277	13.2%	68	12.2%	0.53
Cerebrovascular disease		140	6.7%	65	11.7%	**<0.0001**
PAD		42	2.0%	35	6.3%	**<0.0001**
Atrial fibrillation		57	2.7%	35	6.3%	**<0.0001**
COPD*		24	1.1%	7	1.3%	0.83
Disease of aorta*		20	1.0%	16	2.9%	**0.001**
Malignancy		51	2.4%	17	3.1%	0.41
eGFR (mL/min/1.73 m^2^)		82.36	16.76	49.35	10.37	**<0.0001**
CCS*	Class 1	1345	64.2%	365	65.5%	0.064
	Class 2	636	30.3%	174	31.2%	
	Class 3	69	3.3%	15	2.7%	
	Class 4	46	2.2%	3	0.5%	
NYHA*	I	1765	84.2%	471	84.6%	0.12
	II	279	13.3%	78	14.0%	
	III	23	1.1%	7	1.3%	
	IV	29	1.4%	1	0.2%	
**Initial diagnostic modalities**						
SPECT: n, % for those who underwent SPECT		846/1115	75.9%	269/1115	24.1%	**<0.0001**
Subsequent test	Functional abnormality	381/846	45.1%	127/269	47.2%	0.532
	CT	21/846	2.5%	3/269	1.1%	0.23
	CAG	147/846	17.4%	122/269	45.4%	**<0.0001**
CT: n, % for those who underwent CT		531/618	85.9%	87/618	14.1%	**<0.0001**
Subsequent test	Obstructive CAD	201/531	38.2%	49/87	57.0%	**0.001**
	SPECT	47/531	8.9%	7/87	8.1%	1.00
	CAG	190/531	35.8%	42/87	48.3%	0.031
CAG: n, % for those who underwent CAG		719/920	78.2%	201/920	21.8%	**<0.0001**
Subsequent test	Obstructive CAD	352/719	49.0%	122/201	60.7%	0.0032
	CT	12/719	1.7%	2/201	1.0%	0.75
	SPECT	29/719	4.0%	15/201	7.5%	0.060

Values are number (% of column total, except where indicated) or mean (SD). Proportion of patients in each diagnostic test represents % of patients who underwent each diagnostic test included in the study, with or without decreased eGFR.

P values were calculated from a chi-square test for categorical variables, Continuous variables were expressed as means (standard deviation [SD]). Continuous variables were compared using the Student’s t-test between 2 groups.

^||^Body mass index was calculated as weight in kilograms divided by height in meters squared.

*Potential risk-adjusting variables selected for Cox proportional hazard models. CCS was adjusted for Class 2 or more, and NYHA functional class was adjusted for II or more.

BP = blood pressure, BMI = body mass index, CAD = coronary artery disease, PAD = peripheral artery disease, COPD = chronic obstructive pulmonary disease, eGFR = estimated glomerular rate, CCS = Canadian Circulation Society, NYHA = New York Heart Association, SPECT = single photon emission computed tomography, CT = computed tomography angiography, CAG = coronary angiography.

Statistical analysis of the data was performed by the study biostatistician (YU) using SAS 9.4 software (SAS Institute Inc., Cary, North Carolina). All reported P values were 2-tailed, and P values < 0.05 were considered statistically significant.

## Results

### Patient characteristics

The characteristics of patients in the non-decreased eGFR (N = 2096) and decreased eGFR (N = 557) groups are shown in Table 1. Patients in the decreased eGFR group were older (mean: 70.8 vs. 65.4 years, p < 0.0001) and had a higher prevalence of hypertension (69.3 vs. 54.8%, p < 0.0001), diabetes (32.5 vs. 27.9%, p = 0.032), hyperuricemia (9.0 vs. 4.7%, p = 0.0001), and peripheral (6.3 vs. 2.0%, p < 0.0001) and cerebral vascular diseases (11.7 vs. 6.7%, p < 0.0001).

### Impact of renal dysfunction on diagnostic imaging modalities

The frequency of SPECT between the non-decreased eGFR and decreased eGFR groups was 75.9% and 24.1%, respectively (p < 0.0001), while that of CT was 85.9% and 14.1% (p < 0.0001) and that of CAG was 78.2% and 21.8% (p < 0.0001; Table 1). The patient characteristics with each modality in the decreased eGFR group are presented in Table 2 and are consistent with those in the original J-COMPASS study^15^. In brief, patients who underwent CAG were more likely to be habitual smokers, have peripheral artery or aortic disease, and have high-grade symptoms of angina and dyspnea **(**Table 2 and Supplementary Table 2**)**. After adjusting for confounders, the odds ratio for a higher likelihood to undergo SPECT rather than CT was 1.96 for patients in the decreased eGFR group relative to patients in the non-decreased eGFR group (p < 0.0001), and the odds ratio for a higher likelihood to undergo CAG rather than CT was 1.56 for patients in the decreased eGFR group relative to those in the non-decreased eGFR group (p = 0.0024) **(**Table 3**)**. Renal dysfunction was significantly associated with the choice of initial diagnostic imaging modality.

**Table 2:** Patients characteristics among SPECT, CT and CAG groups in decreased eGFR group.

		SPECT		CT		CAG		P
Age		70.95	9.19	70.34	8.74	70.99	8.73	0.832
Age ≥60 years old		236	87.7%	76	87.4%	181	90.0%	0.690
Female		110	40.9%	36	41.4%	65	32.3%	0.128
Height (cm)		158.89	9.01	158.55	9.05	158.8	8.97	0.955
Weight (Kg)		61.42	10.97	61.36	11.18	61.7	9.99	0.953
BMI (kg/m^2^)		24.25	3.27	24.31	3.47	24.44	3.32	0.824
Systolic Bp (mmHg)		137.42	19.36	138.95	20.84	138.25	19.15	0.787
Diastolic Bp (mmHg)		75.3	11.11	76.8	11.48	76.02	12.62	0.552
Smoking		54	20.1%	16	18.4%	58	28.9%	**0.044**
Hypertension		185	68.8%	62	71.3%	139	69.2%	0.907
Dyslipidemia		114	42.4%	47	54.0%	98	48.8%	0.121
Diabetes		77	28.6%	26	29.9%	78	38.8%	0.056
Hyperuricemia		21	7.8%	9	10.3%	20	10.0%	0.643
Familial history of CAD		24	8.9%	14	16.1%	30	14.9%	0.070
Cerebrovascular disease		33	12.3%	10	11.5%	22	10.9%	0.906
PAD		14	5.2%	1	1.1%	20	10.0%	**0.011**
Atrial fibrillation		22	8.2%	4	4.6%	9	4.5%	0.205
COPD		3	1.1%	1	1.1%	3	1.5%	0.932
Disease of aorta		13	4.8%	2	2.3%	1	0.5%	**0.020**
Malignancy		8	3.0%	0	0.0%	9	4.5%	0.127
eGFR (mL/min/1.73 m^2^)		48.61	9.92	51.58	8.35	49.39	11.58	0.068
CCS	Class 1	214	79.6%	54	62.1%	97	48.3%	**<0.0001**
	Class 2	51	19.0%	32	36.8%	91	45.3%	
	Class 3	4	1.5%	0	0.0%	11	5.5%	
	Class 4	0	0.0%	1	1.1%	2	1.0%	
NYHA	I	240	89.2%	77	88.5%	154	76.6%	**0.004**
	II	27	10.0%	10	11.5%	41	20.4%	
	III	2	0.7%	0	0.0%	5	2.5%	
	IV	0	0.0%	0	0.0%	1	0.5%	

Continuous variables were expressed as means (standard deviation [SD]).

Categorical variables were expressed as numbers and %.

BMI = body mass index, BP = blood pressure, CAD = coronary artery disease, PAD = peripheral artery disease, eGFR = estimated glomerular rate, CCS = Canadian Circulation Society, NYHA = New York Heart Association, SPECT = single photon emission computed tomography, CT = computed tomography, CAG = coronary angiography.

**Table 3:** Factors associated with initial diagnostic modalities and treatment strategies.

Variables	Reference	OR	95%CI		p value
**Factors associated with the use of SPECT**					
eGFR < 60 ml/min/1.73 m^2^	≥60	1.96	1.49	2.59	**<0.0001**
Age ≥60 y.o.	<60	0.85	0.67	1.08	0.18
Female	male	0.94	0.76	1.17	0.57
BMI (kg/m^2^)	1 increase	0.99	0.96	1.03	0.70
Smoking	no	0.84	0.65	1.10	0.20
Hypertension	no	0.87	0.70	1.08	0.20
Dyslipidemia	no	0.91	0.74	1.12	0.35
Diabetes	no	1.10	0.87	1.38	0.45
Hyperuricemia	no	0.85	0.54	1.36	0.50
COPD	no	2.38	1.04	6.46	0.058
Disease of aorta	no	1.08	0.62	1.97	0.79
CCS Class 2 or more	Class 1	0.58	0.45	0.73	**<0.0001**
NYHA II or more	I	0.52	0.37	0.72	**<0.0001**
**Factors associated with the use of CAG**					
eGFR < 60 ml/min/1.73 m^2^	≥60	1.56	1.17	2.08	**0.0024**
Age ≥60 y.o.	<60	1.16	0.90	1.49	0.26
Female	male	0.65	0.52	0.81	**0.0002**
BMI (kg/m^2^)	1 increase	1.02	0.99	1.05	0.26
Smoking	no	1.41	1.09	1.82	**0.009**
Hypertension	no	0.96	0.77	1.20	0.74
Dyslipidemia	no	0.96	0.77	1.18	0.67
Diabetes	no	1.15	0.91	1.46	0.24
Hyperuricemia	no	0.85	0.54	1.34	0.47
COPD	no	3.96	1.78	10.54	**0.0021**
Disease of aorta	no	0.87	0.48	1.65	0.67
CCS Class 2 or more	Class 1	1.57	1.24	1.99	**0.0002**
NYHA II or more	I	0.94	0.70	1.25	0.66
**Factors associated with the escalation of medical therapy relative to the medical therapy**					
CT	SPECT	1.42	1.12	1.81	**0.0045**
CAG	SPECT	2.15	1.68	2.76	**<0.0001**
Age ≥60	<60	1.13	0.90	1.41	0.30
Female	male	0.77	0.63	0.95	**0.016**
BMI (kg/m^2^)	1 increase	1.03	1.00	1.06	**0.027**
Smoking	no	0.88	0.68	1.13	0.31
Hypertension	no	2.12	1.74	2.60	**<0.0001**
Dyslipidemia	no	1.13	0.93	1.39	0.23
Diabetes	no	1.89	1.47	2.43	**<0.0001**
Hyperuricemia	no	0.97	0.61	1.58	0.91
COPD	no	1.24	0.60	2.76	0.57
Disease of aorta	no	2.91	1.59	5.72	**0.0010**
eGFR < 60 ml/min/1.73 m^2^	≥60	1.43	1.10	1.86	**0.0075**
CCS Class 2 or more	Class 1	1.38	1.08	1.77	0.012
NYHA II or more	I	1.14	0.82	1.61	0.44
**Factors associated with the intervention therapy relative to the medical therapy**					
CT	SPECT	1.63	1.20	2.22	**0.0017**
CAG	SPECT	5.30	4.00	7.05	**<0.0001**
Age ≥60	<60	1.67	1.26	2.22	**0.0003**
Female	male	0.34	0.26	0.44	**<0.0001**
BMI (kg/m^2^)	1 increase	1.03	0.99	1.07	0.10
Smoking	no	1.03	0.78	1.37	0.83
Hypertension	no	2.16	1.69	2.76	**<0.0001**
Dyslipidemia	no	2.02	1.59	2.57	**<0.0001**
Diabetes	no	3.89	2.96	5.13	**<0.0001**
Hyperuricemia	no	0.82	0.49	1.39	0.45
COPD	no	1.84	0.87	4.23	0.13
Disease of aorta	no	1.08	0.48	2.48	0.85
eGFR < 60 ml/min/1.73 m^2^	≥60	1.63	1.21	2.21	**0.0015**
CCS Class 2 or more	Class 1	3.48	2.64	4.59	**<0.0001**
NYHA II or more	I	1.21	0.85	1.74	0.30

OR = odds ratio, CI = confidence interval. Abbreviations are same as in Table 2.

### Impact of renal dysfunction on the treatment strategies

**Figure 2:**
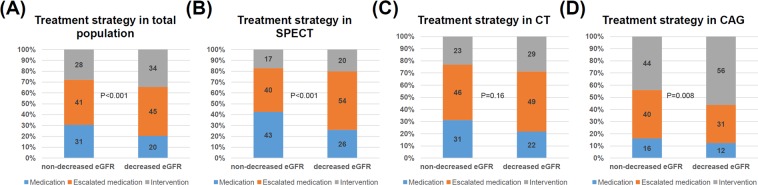
(**A**) The proportion of treatments in non-decreased eGFR and decreased eGFR groups in the entire cohort. (**B**) The proportion of treatments in non-decreased eGFR and decreased eGFR groups in patients underwent SPECT. (**C**) The proportion of treatments in non-decreased eGFR and decreased eGFR groups in patients underwent CT. (**D**) The proportion of treatments in non-decreased eGFR and decreased eGFR groups in patients underwent CAG.

There were differences in treatment strategies between the decreased eGFR and non-decreased eGFR groups in the entire cohort (Fig. 2A) or in patients underwent SPECT or CAG (Fig. 2B–D). After adjusting for confounders, including the initial imaging modality (Table 3), the decrease in eGFR was significantly associated with an escalation of medical therapy (OR 1.43, 95% CI 1.10–1.86, p = 0.0075) and intervention plus an escalation of medical therapy (OR 1.63, 95% CI 1.21–2.21, p = 0.0015).

### Association with CKD stage and the impact of diagnostic imaging modalities on the outcome measures

**Figure 3:**
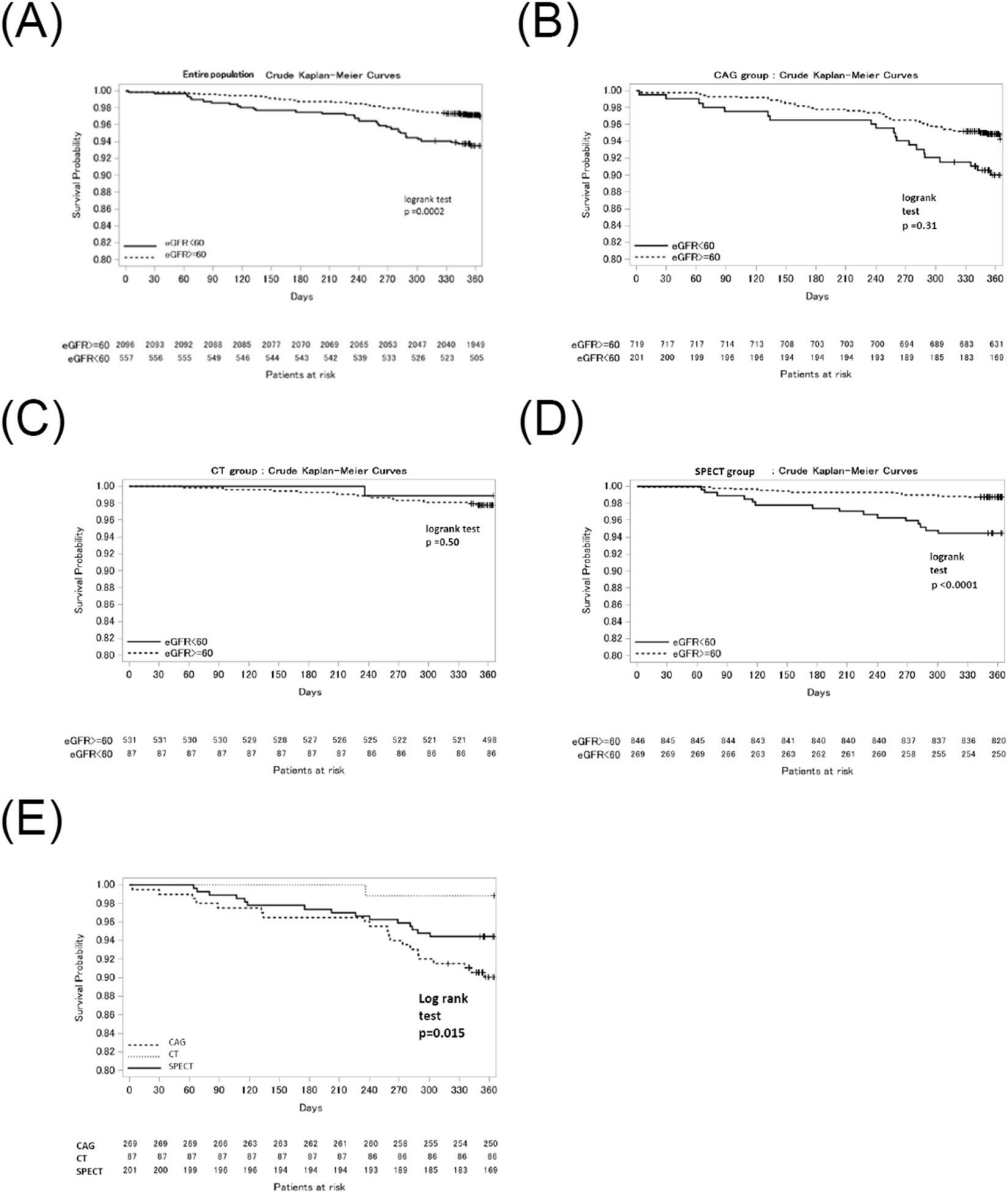
(**A**) Crude Kaplan-Meier curve for MACE. Patients in decreased eGFR group had a poor prognosis compared with patients in non-decreased eGFR group. (**B**) A crude Kaplan-Meier curve for MACE for patients assessed with SPECT. (**C**) Crude Kaplan-Meier curve for MACE for patients assessed with CT. (**D**) Crude Kaplan-Meier curve for MACE for patients assessed with CAG. When stratified by each modality, the crude Kaplan–Meier curves showed the different impacts of renal dysfunction on mortality among the modalities. (**E**) Crude Kaplan-Meier curve for MACE by initial diagnostic modalities in decreased eGFR group.

The median follow-up duration after enrollment was 472 (IQR: 180.93) days, with a 96.2% follow-up rate at 1 year. A crude Kaplan-Meier curve for MACE showed a significantly lower MACE rate among patients in the non-decreased eGFR group (Fig. 3A). After adjusting for confounders, the risk of MACE in the decreased eGFR group was significantly higher relative to that in the non-decreased eGFR group (Table 4). When stratified by modality, the crude Kaplan-Meier curves showed different impacts of a decrease in eGFR on MACE among the modalities; the risk of MACE in the decreased eGFR group relative to that in the non-decreased eGFR group was significant only on SPECT (Fig. 3B–D). After adjusting for confounders, there was a marginally significant interaction between the decrease in eGFR and the prognostic impact of the diagnostic modality. (Table 4). Among patients in the decreased eGFR group, crude Kaplan–Meier curve analysis showed differences in MACEs among the three treatment groups (Fig. 3E). The prevalence of each MACE occurrence in each group is in Supplementary Tables 3 and 4.

**Table 4:** Clinical outcomes of patients in decreased and non-decreased eGFR groups and interaction among diagnostic modalities.

Variables	Non-decreased eGFR group	Decreased eGFR group	Unadjusted				Adjusted			
	N of patients with event (N = 2096)	N of patients with event (N = 557)	HR	95%CI	P value		HR	95%CI	P value	
**Entire cohort**	64/2096 (3.1%)	36/557 (6.5%)	2.16	1.43–3.24	0.0002		1.88	1.23–2.87	0.0036	
**Subgroup**	**N of patients with event/N of patients in subgroup**	**N of patients with event/N of patients in subgroup**	**HR**	**95%CI**	**P value**	**Interaction-p**	**HR**	**95%CI**	**P value**	**Interaction-p**
SPECT	11/946 (1.2%)	15/269 (5.6%)	4.4	2.02–9.57	0.0002	0.0598	3.97	1.80–8.74	0.0006	0.0566
CT	12/531 (2.3%)	1/87 (1.1%)	0.5	0.066–3.88	0.51		0.45	0.059–3.50	0.45	
CAG	41/719 (5.7%)	20/201 (10.0%)	1.78	1.05–3.04	0.0335		1.58	0.91–2.72	0.103	

HR = hazard ratio, CI = confidence interval. Other abbreviations are same as in Table 1.

## Discussion

The main findings of this study were as follows: 1) in patients in the decreased eGFR group, SPECT or CAG was preferred to CT as an initial diagnostic modality by the attending physician; 2) Among the patients who underwent CT or SPECT as an initial test, the prevalence of patients who escalated medication was higher in patients with decreased eGFR compared to patients with non-decreased eGFR. A decrease in eGFR had an independent association with intervention therapy for CAD; 3) On Kaplan-Meier analysis, a decrease in eGFR was not associated with a poor outcome in patients who underwent CT as an initial test, while a decrease in eGFR was associated with poor outcomes in patients who underwent SPECT or CAG as an initial test. After adjusting confounders, a decrease in eGFR was not associated with MACE in patients who underwent CT, while there was a significant association between a decrease in eGFR and MACE in patients who underwent SPECT or CAG as an initial test; and 4) The prognostic impact of a decrease in eGFR tended to be different among the imaging modalities.

### Initial diagnostic imaging and renal dysfunction

SPECT was performed more often than CT in patients with late-stage CKD, probably because of the nephrotoxicity of the intravenous contrast. In addition, the high incidence of abnormal vascular calcification limits the diagnostic value of CT in patients with late-stage CKD^23^. However, CT was performed in some patients with a decrease in eGFR. This might be attributed to the lower cost of CT than SPECT or the fewer numbers of nuclear cardiology facilities in Japan^15^. The decision to perform CAG is often difficult in patients with CKD; however, CAG would be the first choice, without SPECT or CT, in the case of a high pre-test probability. We should balance the risks and potential benefits in patients with CKD^24^. In fact, patients with angina CCS of class 2 or higher were more likely to undergo CAG in the present study. Although the pre-test probability was not determined in the present study, we included the related factors in the multivariate models^25^.

### Subsequent treatment and renal dysfunction

One of the novel findings of the present study is that a decrease in eGFR had an impact on the subsequent treatment strategies. The original J-COMPASS study showed a preference for intervention therapy in patients who underwent CT and CAG compared with those who underwent SPECT, consistently with the findings of the present study^15^. Moreover, renal dysfunction was independently associated with the treatment strategies. One reason for this may be the atherosclerotic burden in patients with renal dysfunction. Another reason may be comorbidities underlying the renal dysfunction, although we performed extensive adjustment for confounding factors.

### Interaction between the impact of renal dysfunction on outcomes and the imaging modalities

The decrease in eGFR was significantly associated with MACE and was an independent predictor of poor outcomes, as previously reported^3^. The novel finding of this study is that we showed the Kaplan-Meier curves in the decreased eGFR and non-decreased eGFR groups according to the diagnostic modality. In the decreased eGFR group, the number of patients using CT as an initial diagnostic modality and the number of MACEs in those patients were very small. This indicated that patients with renal dysfunction were appropriately not considered for CT as an initial diagnostic imaging modality. This was generally consistent with the findings of our original J-COMPASS study^15^, in which the choice of initial imaging modality was linked to the cardiovascular risk. CT and SPECT were suitable for patients with an intermediate pretest probability of the disease^23^. In our study, patients with renal dysfunction were preferably assessed with SPECT and CAG, which explains the differences in the impact of renal dysfunction on the outcomes among modalities. The recent advance in imaging protocol to reduce radiation exposure and decrease iodine dose or isotope tracer dose in coronary CT angiography or SPECT may affect the choice of diagnostic modalities. Especially, ultra-low-dose contrast coronary CT protocol^26^ which can be performed with optimal image quality minimizing the risk for radiation exposure and contrast-induced nephrotoxicity might be feasible for CKD patients. Because the present study is not a randomized controlled study, we could not evaluate the validity of the criteria for selecting diagnostic tests or the appropriateness of the treatment planning. In patients in the decreased eGFR group, the outcomes were getting worse in the following order: CT, SPECT, and CAG (Fig. 3E) without adjustment for confounders. However, because of the marginal significance of interaction and the small number in the CT group with renal dysfunction, we could not definitely conclude that the negative prognostic impact of renal dysfunction could be reduced by appropriately choosing the initial imaging modality. Instead, we could speculate that the optimal diagnostic test and treatment for each patient were determined on the basis of the clinical characteristics and renal function of each patient. This is supported by the substantial number of patients of the decreased eGFR group undergoing CAG and CT.

### Link between diagnostic modalities and outcomes in patients with renal dysfunction

Obviously, it is not the diagnostic test itself but the subsequent treatment based on the findings of the diagnostic test that improves clinical outcomes in patients with suspected CAD. Douglas *et al*. reported that patients in the CT group underwent revascularization more frequently than did those in the initial functional test group^5^; however, the CT group did not reduce incidence of outcome compared to functional test group. In line with the evidence from this large randomized trial, patients with CT and CAG were more likely to undergo revascularization than were those with SPECT in the original J-COMPASS study^15^. In the present study, we showed that renal dysfunction was an independent factor associated with optimal medical treatment and revascularization, regardless of the diagnostic modality. The study was not designed to assess coronary revascularization or medical treatment and its appropriateness and effect on outcomes. In fact, we did not include treatment, which was dependent on the initial and, sometimes, subsequent diagnostic tests, in the adjustment for the outcome measures. The impact of the treatment after initial functional imaging on the outcomes is under investigation in an ongoing ISCHEMIA trial (International Study of Comparative Health Effectiveness with Medical and Invasive Approaches, NCT01471522), wherein invasive therapy vs. medical therapy is randomized in patients with functional ischemia; however, CT was not performed in the decreased eGFR group. Despite the interaction observed on initial diagnostic imaging, our study showed worse outcomes in the decreased eGFR group.

One of the imaging modalities for CAD not included in the preset study is positron emission tomography (PET). Quantitative approaches that measure MBF with PET identify multi-vessel CAD^27^. In addition, it is well known that patients with CKD with microvascular dysfunction which can be measured by PET have poor outcome while SPECT often underestimates microvascular dysfunction^27^, although it remains to be underutilized in clinical practice.

### Study limitations

There are some limitations in the present study. We did not analyze or collect information about why and how patient treatment decisions were made, including the findings of the diagnostic tests, cost^28^, coronary flow reserve in CAG, and the administered drugs. We did not verify the quality of the diagnostic imaging modalities at each participating center, although all centers had high-end diagnostic facilities. In addition, we did not collect the data on what constituted medical therapy or the escalation of medical therapy, nor on the parameters for the escalation of the medical therapy. In the analyses, we did not perform the analysis on whether the subsequent diagnostic test affects the MACE rates and subsequent treatment strategy, along with the findings of the initial diagnostic test. Further, we did not collect data on the indication for and type of medication used during escalation of medical therapy. Thus, further studies are needed to evaluate the relationship between the findings of diagnostic tests and subsequent tests and treatments. It is possible that there remain unmeasured confounders that affect the choice of modalities, treatments, and outcomes, although we conducted extensive statistical adjustment for the measured confounders. Tracking of the outcomes with and without revascularization would be helpful in conjunction with the medical treatment. The use of CT is increasing annually, and it is frequently being used for screening for CAD in Japan^29,30^. Therefore, we should be careful while generalizing the results of the present study. Finally, the impact of renal dysfunction on long-term prognosis is still unclear in our study population and needs to be elucidated.

## Conclusions

Renal dysfunction was found to be associated with the choice of imaging modality for CAD, as well as with the treatment strategy for CAD. The prognostic impact of renal dysfunction tended to be different among the imaging modalities.

## Supplementary information

Supplementary materials

## Data Availability

The datasets generated and/or analyzed during the current study are available from the corresponding author upon reasonable request.

## References

[1] Wang H (2015). Global, regional, and national life expectancy, all-cause mortality, and cause-specific mortality for 249 causes of death, 1980–2015: a systematic analysis for the Global Burden of Disease Study. The Lancet.

[2] Saran, R. *et al*. US Renal Data System 2017 Annual Data Report: epidemiology of kidney disease in the United States. *American journal of kidney diseases: the official journal of the National Kidney Foundation* (2017). 10.1053/j.ajkd.2018.01.002 PMC6593155 29477157

[3] Sarnak MJ (2003). Kidney disease as a risk factor for development of cardiovascular disease: a statement from the American Heart Association Councils on Kidney in Cardiovascular Disease, High Blood Pressure Research, Clinical Cardiology, and Epidemiology and Prevention. Circulation.

[4] Ix JH (2003). Association between Renal Insufficiency and Inducible Ischemia in Patients with Coronary Artery Disease: The Heart and Soul Study. Journal of the American Society of Nephrology.

[5] Douglas PS (2015). Outcomes of anatomical versus functional testing for coronary artery disease. The New England journal of medicine.

[6] Atkinson P (2011). Predictive value of myocardial and coronary imaging in the long-term outcome of potential renal transplant recipients. International journal of cardiology.

[7] Cho I (2010). Coronary atherosclerosis detected by coronary CT angiography in asymptomatic subjects with early chronic kidney disease. Atherosclerosis.

[8] Dewey M (2016). Evaluation of computed tomography in patients with atypical angina or chest pain clinically referred for invasive coronary angiography: randomised controlled trial. Bmj.

[9] Furuhashi T (2013). The predictive value of chronic kidney disease for assessing cardiovascular events under consideration of pretest probability for coronary artery disease in patients who underwent stress myocardial perfusion imaging. The international journal of cardiovascular imaging.

[10] Joki N (2014). Myocardial perfusion imaging for predicting cardiac events in Japanese patients with advanced chronic kidney disease: 1-year interim report of the J-ACCESS 3 investigation. Eur J Nucl Med Mol Imaging.

[11] Jug B (2013). Diagnostic performance of computed tomographic coronary angiography in patients with end-stage renal disease. Coronary artery disease.

[12] Mudrick DW (2013). Patterns of stress testing and diagnostic catheterization after coronary stenting in 250 350 medicare beneficiaries. Circulation. Cardiovascular imaging.

[13] Williams KA (2009). Chronic kidney disease, SPECT, and coronary angiography: “head of gold and feet of clay?”. Journal of nuclear cardiology: official publication of the American Society of Nuclear Cardiology.

[14] Yiu KH (2011). Prognostic value of renal dysfunction for the prediction of outcome versus results of computed tomographic coronary angiography. Am J Cardiol.

[15] Yamauchi T (2012). Optimal initial diagnostic strategies for the evaluation of stable angina patients: a multicenter, prospective study on myocardial perfusion imaging, computed tomographic angiography, and coronary angiography. Circulation journal: official journal of the Japanese Circulation Society.

[16] Matsuo S (2009). Revised equations for estimated GFR from serum creatinine in Japan. American journal of kidney diseases: the official journal of the National Kidney Foundation.

[17] Austen WG (1975). A reporting system on patients evaluated for coronary artery disease. Report of the Ad Hoc Committee for Grading of Coronary Artery Disease, Council on Cardiovascular Surgery, American Heart Association. Circulation.

[18] Meijboom WB (2006). Pre-operative computed tomography coronary angiography to detect significant coronary artery disease in patients referred for cardiac valve surgery. Journal of the American College of Cardiology.

[19] Cerqueira MD (2002). Standardized myocardial segmentation and nomenclature for tomographic imaging of the heart. A statement for healthcare professionals from the Cardiac Imaging Committee of the Council on Clinical Cardiology of the American Heart Association. Circulation.

[20] Naya M (2018). Long-term events after physician-referred initial tests by myocardial perfusion imaging or computed tomography coronary angiography in patients with suspected coronary artery disease. Coronary artery disease.

[21] Campeau L (1976). Letter: Grading of angina pectoris. Circulation.

[22] Editorial: Major changes made by Criteria Committee of the New York Heart Association. *Circulation* **49**, 390 (1974). 10.1161/01.cir.49.3.390 4813168

[23] Genders TS (2015). The optimal imaging strategy for patients with stable chest pain: a cost-effectiveness analysis. Annals of internal medicine.

[24] Choi HY, Park HC, Ha SK (2014). How do We Manage Coronary Artery Disease in Patients with CKD and ESRD?. Electrolyte & blood pressure: E & BP.

[25] Genders TS (2012). Prediction model to estimate presence of coronary artery disease: retrospective pooled analysis of existing cohorts. Bmj.

[26] Komatsu S (2013). Coronary computed tomography angiography using ultra-low-dose contrast media: radiation dose and image quality. The international journal of cardiovascular imaging.

[27] Schindler TH, Schelbert HR, Quercioli A, Dilsizian V (2010). Cardiac PET imaging for the detection and monitoring of coronary artery disease and microvascular health. JACC. Cardiovascular imaging.

[28] Mark DB (2016). Economic Outcomes With Anatomical Versus Functional Diagnostic Testing for Coronary Artery Disease. Annals of internal medicine.

[29] Kasai T (2017). Trends and Perspectives of Stress Myocardial Perfusion Imaging in Japan. Ann Nucl Cardiol..

[30] Cho SG (2017). Myocardial Perfusion Imaging in East and West: Challenge or Chance?. Ann Nucl Cardiol..

