# Supplementary material for: Impact of renal dysfunction on the choice of diagnostic imaging, treatment strategy, and outcomes in patients with stable angina
Source: Sci Rep. 2019 May 27;9:7882. doi: 10.1038/s41598-019-44371-4 (PMC6536514; doi:10.1038/s41598-019-44371-4)
Supplement: Supplementary file 1 — Supplementary materials [file 41598_2019_44371_MOESM1_ESM.docx]

**Supplementary materials for**

Impact of renal dysfunction on choice of diagnostic imaging, treatment strategy, and outcomes in patients with stable angina

Takao Kato; Yukari Uemura; Masanao Naya; Mitsuru Momose; Naoya Matsumoto; Eriko Suzuki; Satoshi Hida; Takatomo Nakajima; Takao Yamauchi; Nagara Tamaki.

**Include 4 supplementary tables and Appendix.**

**Supplementary Table 1. Study population and excluded patients**

|  | MPI | CT | CAG | Total |
| --- | --- | --- | --- | --- |
| Full cohort | 1205 | 625 | 950 | 2780 |
|  |  |  |  |  |
| Dialysis | 32 | 2 | 24 | 58 |
| Undetermined values | 58 | 5 | 6 | 69 |
|  |  |  |  |  |
| Study population | 1115 | 618 | 920 | 2653 |
|  |  |  |  |  |

MPI= myocardial perfusion imaging, CTA=computed tomographic angiography, CAG=coronary angiography

**Supplementary Table 2.** Patient characteristics in each modality

|  | | SPECT | | | | CT | | | | CAG | | | |
| --- | --- | --- | --- | --- | --- | --- | --- | --- | --- | --- | --- | --- | --- |
|  |  | （n = 1115） | | | | （n = 618） | | | | （n = 920） | | | |
|  |  | Decreased eGFR group (n=269) | | Non-decreased eGFR group (n=846) | | Decreased eGFR group (n=87) | | Non-decreased eGFR group (n=531) | | Decreased eGFR group (n=201) | | Non-decreased eGFR group (n=719) | |
| Age | | 70.95 | 9.19 | 64.99 | 10.45 | 70.34 | 8.74 | 65.33 | 10.41 | 70.99 | 8.73 | 66.13 | 10.4 |
| Age≥60 years old | | 236 | 87.7% | 578 | 68.3% | 76 | 87.4% | 381 | 71.8% | 181 | 90.0% | 517 | 71.9% |
| Female | | 110 | 40.9% | 389 | 46.0% | 36 | 41.4% | 252 | 47.5% | 65 | 32.3% | 247 | 34.4% |
| Height (cm) | | 158.89 | 9.01 | 159.23 | 9.18 | 158.55 | 9.05 | 158.54 | 8.83 | 158.8 | 8.97 | 159.99 | 8.85 |
| Weight (Kg) | | 61.42 | 10.97 | 60.4 | 12.05 | 61.36 | 11.18 | 60.24 | 11.37 | 61.7 | 9.99 | 62.2 | 11.47 |
| BMI (kg/m^2^) | | 24.25 | 3.27 | 23.72 | 3.66 | 24.31 | 3.47 | 23.87 | 3.38 | 24.44 | 3.32 | 24.19 | 3.36 |
| Systolic Bp (mmHg) | | 137.42 | 19.36 | 137.17 | 19.7 | 138.95 | 20.84 | 139.2 | 19.68 | 138.25 | 19.15 | 135.44 | 18.36 |
| Diastolic Bp (mmHg) | | 75.3 | 11.11 | 78.91 | 12.08 | 76.8 | 11.48 | 78.24 | 12.56 | 76.02 | 12.62 | 77.34 | 11.36 |
| Smoking | | 54 | 20.1% | 174 | 20.6% | 16 | 18.4% | 118 | 22.2% | 58 | 28.9% | 228 | 31.7% |
| Hypertension | | 185 | 68.8% | 435 | 51.4% | 62 | 71.3% | 297 | 55.9% | 139 | 69.2% | 417 | 58.0% |
| Dyslipidemia | | 114 | 42.4% | 395 | 46.7% | 47 | 54.0% | 262 | 49.3% | 98 | 48.8% | 349 | 48.5% |
| Diabetes | | 77 | 28.6% | 238 | 28.1% | 26 | 29.9% | 137 | 25.8% | 78 | 38.8% | 209 | 29.1% |
| Hyperuricemia | | 21 | 7.8% | 34 | 4.0% | 9 | 10.3% | 27 | 5.1% | 20 | 10.0% | 38 | 5.3% |
| Familial history of CAD | | 24 | 8.9% | 107 | 12.6% | 14 | 16.1% | 81 | 15.3% | 30 | 14.9% | 89 | 12.4% |
| Cerebrovascular disease | | 33 | 12.3% | 65 | 7.7% | 10 | 11.5% | 27 | 5.1% | 22 | 10.9% | 48 | 6.7% |
| PAD | | 14 | 5.2% | 16 | 1.9% | 1 | 1.1% | 5 | 0.9% | 20 | 10.0% | 21 | 2.9% |
| Atrial fibrillation | | 22 | 8.2% | 21 | 2.5% | 4 | 4.6% | 16 | 3.0% | 9 | 4.5% | 20 | 2.8% |
| COPD | | 3 | 1.1% | 9 | 1.1% | 1 | 1.1% | 5 | 0.9% | 3 | 1.5% | 10 | 1.4% |
| Disease of aorta | | 13 | 4.8% | 9 | 1.1% | 2 | 2.3% | 2 | 0.4% | 1 | 0.5% | 9 | 1.3% |
| Malignancy | | 8 | 3.0% | 19 | 2.2% | 0 | 0.0% | 11 | 2.1% | 9 | 4.5% | 21 | 2.9% |
| eGFR(mL/min/1.73m^2^) | | 48.61 | 9.92 | 82.17 | 16.65 | 51.58 | 8.35 | 83.32 | 17.57 | 49.39 | 11.58 | 81.86 | 16.25 |
| CCS | Class1 | 214 | 79.6% | 647 | 76.5% | 54 | 62.1% | 325 | 61.2% | 97 | 48.3% | 373 | 51.9% |
|  | Class2 | 51 | 19.0% | 188 | 22.2% | 32 | 36.8% | 167 | 31.5% | 91 | 45.3% | 281 | 39.1% |
|  | Class3 | 4 | 1.5% | 8 | 0.9% | 0 | 0.0% | 15 | 2.8% | 11 | 5.5% | 46 | 6.4% |
|  | Class4 | 0 | 0.0% | 3 | 0.4% | 1 | 1.1% | 24 | 4.5% | 2 | 1.0% | 19 | 2.6% |
| NYHA | I | 240 | 89.2% | 782 | 92.4% | 77 | 88.5% | 420 | 79.1% | 154 | 76.6% | 563 | 78.3% |
|  | II | 27 | 10.0% | 61 | 7.2% | 10 | 11.5% | 81 | 15.3% | 41 | 20.4% | 137 | 19.1% |
|  | III | 2 | 0.7% | 3 | 0.4% | 0 | 0.0% | 11 | 2.1% | 5 | 2.5% | 9 | 1.3% |
|  | IV | 0 | 0.0% | 0 | 0.0% | 0 | 0.0% | 19 | 3.6% | 1 |  | 10 | 1.4% |

Values are number (%), or mean (SD).

Continuous variables were expressed as means (standard deviation [SD])..

BP=blood pressure, BMI=body mass index, CAD=coronary artery disease, PAD=peripheral artery disease, COPD=chronic obstructive pulmonary disease, eGFR=estimated glomerular rate, CCS=Canadian Circulation Society, NYHA= New York Heart Association, SPECT=single photon emission computed tomography, CT=computed tomography angiography, CAG=coronary angiography.

**Supplementary Table 3.**

|  | Non-decreased eGFR | | | | Decreased eGFR | | | |
| --- | --- | --- | --- | --- | --- | --- | --- | --- |
|  | With MACE | | Without MACE | | With MACE | | Without MACE | |
|  | N | % | N | % | N | % | N | % |
| SPECT | 11 | 1.3% | 835 | 98.7% | 15 | 5.6% | 254 | 94.4% |
| CT | 12 | 2.3% | 519 | 97.7% | 1 | 1.1% | 86 | 98.9% |
| CAG | 41 | 5.7% | 678 | 94.3% | 20 | 10.0% | 181 | 90.0% |

eGFR=estimated glomerular filtration rate, MACE=major adverse cardiac event

**Supplementary Table 4.**

|  |  | Non-decreased eGFR | Decreased eGFR |
| --- | --- | --- | --- |
| SPECT | Death | 1 | 4 |
|  | AMI | 8 | 7 |
|  | HF | 1 | 4 |
|  | Late revascularization | 1 | 0 |
| CT | Death | 4 | 0 |
|  | AMI | 7 | 0 |
|  | HF | 1 | 1 |
|  | Late revascularization | 0 | 0 |
| CAG | Death | 7 | 7 |
|  | AMI | 20 | 8 |
|  | HF | 5 | 2 |
|  | Late revascularization | 9 | 3 |

AMI=acute myocardial infarction, HF=heart failure

**Appendix**

List of participating hospitals for the J-COMPASS and Institutional review board of:

Aichi Medical University, Anjo Kosei Hospital, Cardiovascular Hospital of Central Japan, Eastern Japan Medical Center, Ebara Hospital, Ebina General Hospital, Enshu Hospital, Furano Hospital, Gifu Prefectural General Medical Center, Gunma Cardiovascular Center, Higashiosaka City General Hospital, Hokkaido University Hospital, Hokko Memorial Hospital, Hyogo College Of Medicine, Iizuka Hospital, Kagoshima City Hospital, Kansai Rosai Hospital, Kihara Junkankinaika, Kitano Hospital, Kobe City Medical Center West Hospital, Konan Saint Hill Hospital, Kosei Hospital, Kyoto University Hospital, Maizuru Kyosai Hospital, Matsuyama Cardiovascular Medical Center, Mie Heart Center, Minamiosaka Hospital, Mitsubishi Kyoto Hospital, Nagoya Tokusyukai General Hospital, National Hospital Organization Hokkaido Cancer Center, National Hospital Organization Kagoshima Medical Center, National Hospital Organization Kumamoto Medical Center, National Hospital Organization Kyoto Medical Center, National Hospital Organization Kyushu Medical Center, National Hospital Organization Sagamihara National Hospital, National Hospital Organization Zentsuji National Hospital, Nihon University Itabashi Hospital, Nippon Medical School Chiba Hokusoh Hospital, Nozaki Tokushukai Hospital, Osaka City General Hospital, Saiseikai Futsukaichi Hospital, Saiseikai Kumamoto Hospital, Saiseikai Kurihashi Hospital, Saiseikai Matsuyama Hospital, Saiseikai Senri Hospital, Saiseikai Yokohamashi Tobu Hospital, Saitama Cardiovascular and Respiratory Center, Saitama Medical University International Medical Center, Sakakibara Memorial Hospital, Sakurakai Takahashi Hospital, Sapporo Medical University Hospital, Sendai Cardiovascular Center, Shiga University of Medical Science Hospital, Shinko Kakogawa Hospital, Shin-Koga Hospital, Shinmatsudo Central General Hospital, Steel Memorial Muroran Hospital, Surugadai Nihon University Hospital, Takamatsu Red Cross Hospital, Takase-Clinic, Takeshi Kondo, Tenri Hospital, Toho University Ohashi Medical Center, Toho University Omori Medical Center, Tokai University Hospital, Tokushima Prefectural Central Hospital, Tokushima Red Cross Hospital, Tokyo KoseiNenkin Hospital, Tokyo Medical University Hachioji Medical Center, Tokyo Medical University Hospital, Tokyo Medical University Ibaraki Medical Center, Tokyo Metropolitan Tama Medical Center, Tokyo Saiseikai Central Hospital, Tokyo Women’s Medical University Hospital, Tominaga Hospital, Toyooka Hospital, Tsurumi Junkanki Clinic, Ube Industry Central Hospital, Wakayama Medical University Hospital, Yokohama Asahi Chuo General Hospital, Yokohama City University Medical center, and Yokohama General Hospital.
